# The Parametric Model of the Human Mandible Coronoid Process Created by Method of Anatomical Features

**DOI:** 10.1155/2015/574132

**Published:** 2015-05-06

**Authors:** Nikola Vitković, Jelena Mitić, Miodrag Manić, Miroslav Trajanović, Karim Husain, Slađana Petrović, Stojanka Arsić

**Affiliations:** ^1^Faculty оf Mechanical Engineering, University of Niš, Aleksandra Medvedeva 14, 18000 Niš, Serbia; ^2^University of Qadisiya, Diwaniya, Iraq; ^3^Faculty of Medicine, University of Niš, Dr. Zorana Đinđića 81 Boulevard, 18000 Niš, Serbia

## Abstract

Geometrically accurate and anatomically correct 3D models of the human bones are of great importance for medical research and practice in orthopedics and surgery. These geometrical models can be created by the use of techniques which can be based on input geometrical data acquired from volumetric methods of scanning (e.g., Computed Tomography (CT)) or on the 2D images (e.g., X-ray). Geometrical models of human bones created in such way can be applied for education of medical practitioners, preoperative planning, etc. In cases when geometrical data about the human bone is incomplete (e.g., fractures), it may be necessary to create its complete geometrical model. The possible solution for this problem is the application of parametric models. The geometry of these models can be changed and adapted to the specific patient based on the values of parameters acquired from medical images (e.g., X-ray). In this paper, Method of Anatomical Features (MAF) which enables creation of geometrically precise and anatomically accurate geometrical models of the human bones is implemented for the creation of the parametric model of the Human Mandible Coronoid Process (HMCP). The obtained results about geometrical accuracy of the model are quite satisfactory, as it is stated by the medical practitioners and confirmed in the literature.

## 1. Introduction

Geometrical models of human bones are of great importance in today's medicine, as well as in anthropology and other related disciplines. Computer-Assisted Surgery (CAS) is one of the most common applications of computer generated geometrical models, as stated by Adams et al. in [1]. The application of geometrically precise models enables surgeons to properly prepare and perform interventions with use of suitable computer software tools and/or other techniques, and it lessens the possibility of error occurrence. The comparison of conventional methods and CAS is presented in [2] by Bäthis et al., where the Total Knee Arthroplasty (TKA) process is shown. Based on the facts stated in [2], we can conclude that the new technique of performing surgical procedures, that is, surgical interventions, may significantly improve both the quality of the procedure itself and the patients' convalescence.

The preoperative planning of surgical procedures and interventions is an important part of CAS. Preoperative planning most often implies the use of suitable human organ models in specific software which enables a surgeon to plan the course of surgical procedure up to a specific level defined by limitations of the applied software. The application of preoperative planning in the case of mandible reconstruction is presented in [3] by Essig et al. and in [4] by Chapius et al.

Geometrical models of human bones created as aforementioned may find their use in the area of virtual anthropology (VA). VA is an area which extends comparative morphology but implies introducing and establishing interconnection among anthropology, mathematics, statistics, engineering, and other areas of science and technology directed to digitalization of observed objects fossil specimens (e.g., bones). Students of anthropology, as well as practitioners, can learn necessary information from precise geometrical models of bones. A detailed description of virtual anthropology, along with the description of methods and techniques applied in this area of research, is provided in [5] by Weber and in [6] by Benazzi et al., in the case of mandible reconstruction.

The basic mandible reconstruction can be performed based on volumetric methods of scanning (Computed Tomography (CT), Magnetic Resonance Imaging (MRI), etc.) as presented in [5, 6] as well as by direct methods of Computer Aided Design (CAD) [5].

Volumetric methods of scanning imply the use of scanner to form volumetric model by the application of different techniques and methods described in detail in [6]. Basically, this is the reverse engineering procedure and contains multiple actions. The first step is to form 2D image (slice) of human body on volumetric scanner. By superposition of provided slices a volumetric image of scanned object (patient) is formed, comprised of volumetric elements (voxels). By further process of segmentation, a detailed bonding of anatomical entities along the whole volume of the scanned model is performed, as shown in [7] by Archipa et al. Segmentation can be a very complex process, and lots of studies have been done to solve problems with feature extraction, like it is described in [8] by Huang et al. for the automatic extraction of the vertebral column from the SPECT (Single-Photon Emission Computed Tomography) scan of the whole body. Through an adequate process of volumetric rendering, a reconstructed 3D model of the scanned object is acquired [9]. Volumetric rendering implies shading of projected 3D scalar field (cloud of points) onto 2D, that is, the computer screen, and it is applied in various areas of computer graphics, as described in [10] by Li et al. Based on created 3D scalar field (initially segmented volumetric model comprised of voxels), by application of an adequate algorithm, such as marching cubes algorithm which is described in [11] by Lorensen and Cline, a polygonal model (mesh) of scanned object can be constructed. Polygonal model can be further used in CAD software packages for creation of surface and volume models, as presented in [12] by Tufegdžić et al. Such models are constructed based on geometry of a specific patient, and, thus, they can be used to create implants and fixators adjusted to the patient, in preoperative planning, intraoperational navigation, and so forth.

Direct modeling implies the creation of models by use of technical elements of CAD software packages. This sort of modeling does not use scanned models; modeling is performed based on information in the form of images, instructions, and presentational models (models of bone and joint system). The geometrical and anatomical accuracy of the models created by the application of these methods is less than the accuracy of models created by the reverse engineering methods. Thus created models can be used for training students and medical practitioners, for the creation of presentational models by use of additive technologies, which are described in [13] by Salmoria et al. and in all other applications where there is no need for geometrical models of great precision.

Creation of geometrical models of human bones, mandible included, can be performed based on predictive models. Predictive models (most often parametric models) are models whose geometry and topology can be adjusted to a specific patient, based on specific parameters (most commonly morphometric, but also others, such as height and weight). Morphometric parameters are acquired from 2D images (X-ray) or from volumetric models obtained by a volumetric scanning method (CT, MRI) [5, 6]. Such models can be very precise, if a number of parameters are adequate and the model structure itself is well chosen (e.g., containing parametric surfaces like NURBS surfaces). These models can be used for many purposes: creation of implants and fixators, preoperative planning, creating geometrical models of the missing parts of bones, and so forth.

It is important to mention that predictive models are created not only for the human bones, but also for the other parts of the human body (or even whole body). In [14] by Li et al. prediction of the deformations and movements of body organs/tissues and skeletal structures using patient-specific nonlinear biomechanical modeling from whole body CT image registration is presented. Besides volumetric internal scanning methods (CT, MRI, etc.), there is a possibility of creating predictive human body or parts of the body models based on the various types of the 3D measurements, like it is shown in [15] by Wuhrer and Shu and also by Leong et al. in [16]. These research studies enable feature extraction and prediction of the shape of the human body's anatomical section, as demonstrated in the example of reconstruction of the human torso in [16]. Deformable statistical whole body model which can be adapted to the single 2D image is presented in [17] by Chen et al. Model presented in [17] can be applied for the creation of whole body meshes or clothed 3D meshes for different people, neither of which appears in the training dataset.

In this paper Method of Anatomical Features (MAF), which was introduced in [18] by Vitković et al. and in [19] by Majstorovic et al., is implemented for the creation of the parametric (predictive) model of the Human Mandible Coronoid Process (HMCP). The MAF was originally applied for the development of the parametric and surface models of the human femur and tibia, and the results are quite satisfactory, as presented in [18, 19]. The main objective of this research is to show that MAF can be applied for other types of human bones, not just for the long bones. The HMCP was chosen because of its complex geometric and topological properties, and it is adequate anatomical section for the creation of prototype (test) parametric model. MAF was tested on prototype model and the results are more than promising. The research will be continued for the creation of the parametric model of the whole human mandible, so that the geometrical and anatomical correctness of the whole model can be confirmed.

## 2. Material and Methods

For the geometry analysis of the human mandible, ten (10) mandible samples were scanned (input training set). The samples were made by 64-slice CT (MSCT) (Aquilion 64, Toshiba, Japan), according to the standard protocol recording: radiation of 120 kVp, current of 150 mA, rotation time of 0.5 s, exposure time of 500 ms, rotation time 0.5 s, thickness of 0.5 mm, image resolution 512 × 512 px, and pixel size approximately 0.36–0.42 mm, 16 bits allocated and stored. The samples came from Serbian adults, intentionally including different gender and age: six male samples aged 25–67 and four women samples aged 22–72, of different height and weight, which have been previously scanned (because of trauma or some disease). It was assumed that this diverse set of samples could present quite a diverse morphology of the very same bone. These samples are used for the creation of the parametric model of the human mandible. The process of creation of parametric model for femur and tibia by using MAF is presented in [18, 19] in detail, but here the short introduction of the method is shown.

**Figure 1:**
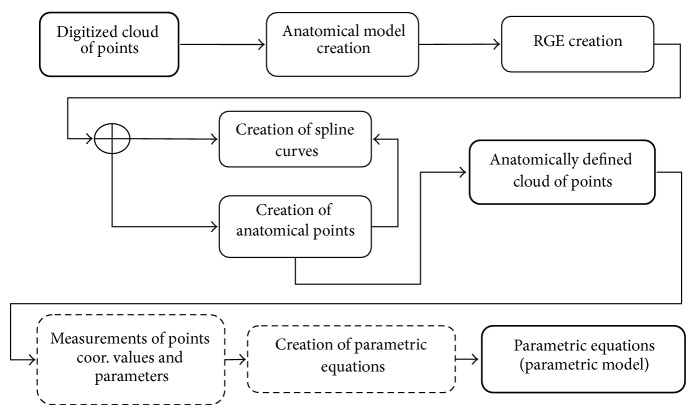
Scheme of the MAF method applied for the creation of parametric model of the human bone.

The process of creation of parametric model of the human bone (MAF method) is presented in Figure 1 and it contains several steps:

- Creation of anatomical model, morphologically and anatomically defined descriptive model of human bone. This model defines where some anatomical feature on the physical model of the bone is and its morphometrical and geometrical relations to other anatomical features.
- RGE creation. The basic prerequisite for successful reverse modelling of a human bone's geometry is identification of referential geometrical entities (RGEs). Usually, these RGEs include characteristics, points, directions, planes, and views, as presented in [18, 19].
- Creation of spline curves. Spline curves are created by the use of RGEs and additional geometry. How curves are created depends on the shape of anatomical feature and its relation to other anatomical features.
- Creation of anatomical points. Anatomical points can be created on spline curves and/or anatomical landmarks. Anatomical points created on spline curves can be positioned in two distinctive ways. First, they can be distributed evenly on the curve or they can be positioned in correspondence to some anatomical landmark. For example, anatomical point can be placed on gnathion of mandible.
- Measurement of anatomical points coordinates values for defined number of specimens. Values of coordinates are measured on each sample of mandible model in 3D. Values of morphometric parameters (defined in the step of anatomical model creation) are measured on the same 3D models.
- The measured data which is processed in mathematical software by using multilinear regression as the tool for statistical analysis.
- Parametric equations (functions) which define relations between morphometric parameters and coordinate values. The created parametric model which consists of a set of parametric equations is a predictive model. This means that, for every next patient, it is enough to measure the same morphometric parameters on scanned mandible and to calculate coordinates of points. The resulting model is cloud of calculated anatomical points which can be imported in any CAD software (e.g., CATIA).

The whole process of the creation of parametric model of the HMCP is presented in the next section of the paper.

### 2.1. Anatomical Model of Human Mandible

Anatomical model is morphologically and anatomically defined descriptive model of human bone. This model defines where some anatomical feature on the real bone is and its morphometrical and geometrical relations to other anatomical features [18].

**Figure 2:**
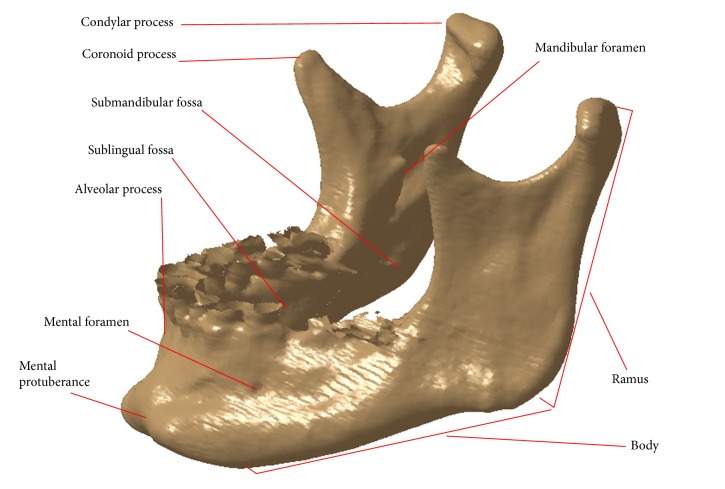
Anatomy of human mandible.

Lower jaw (mandible) is the biggest and the most massive face bone, which is connected with skull bones through the temporomandibular joint. It represents the biggest odd bone of the face or the viscecranial bone, which participates in construction of the only mobile head joint. It consists of mandible body and two rami as described in [20, 21] by Juodzbalys et al. and presented in Figure 2.

Mandible body (Latin: corpus mandibulae) is of horseshoe shape and represents its horizontal part. It consists of two sides (external and internal) and two edges, alveolar part of the mandible which corresponds with inferior dental arch (Latin: arcus alveolaris) and a lower edge or mandible basis (Latin: basis mandibulae).

Ramus is approximately of a rectangle shape which is located upward and backward in relation to mandible body with which it forms an angle of 90°–140°, most commonly 120°–130°. It has two sides, external and internal, and four edges, upper, lower, anterior, and posterior. The upper edge has two processes: coronoid process (Latin: processus coronoideus) and condylar process (Latin: processus condylaris).

### 2.2. Referential Geometrical Entities (RGEs)

In reverse modeling of geometry of human mandible, it is crucial to both determine directions and projections of bone parts or the whole bone and establish rules for creation of all directions and views, which should be precisely used. For better orientation, we use several orientational lines and planes in dental medicine.

- Medial line, a line which passes vertically between central incisors and which mainly divides the face into two equal parts.
- Sagittal (central) plane, which passes through the body and divides it into equal halves right and left.
- Frontal plane, which passes through the body in direction left-right (parallel with the forehead) and divides the body into anterior and posterior parts.
- Transversal (horizontal) planes, positioned horizontally, which when in basic anatomical position pass through the body parallel with the ground.

The basic prerequisite to successfully perform reverse modeling of human bone geometry is identification of referential geometrical entities (RGEs). RGEs include characteristic points, directions, planes, and views. Other elements of bone curve and surface geometry will be defined with reference to RGEs. To create precise geometry of a human bone, a set of primary RGEs should be minimized. Geometrical limitations and relations should be based on a minimal set of primary RGEs. This is an approach for a successful parameterization of human bone geometry. All mentioned planes and lines are RGEs of the human mandible geometrical model.

The ability to create anatomical landmarks as geometrical elements on 3D human bone models has a significant role and a vast potential for bone reconstruction after innate defects, illnesses, and traumas. Anatomical landmarks are defined on each polygonal model of human mandible of the acquired samples. They can be defined relative to the RGEs, they can be defined as RGEs, or they can be created on the support geometry which is relative to RGEs (e.g., spline curves).

**Figure 3:**
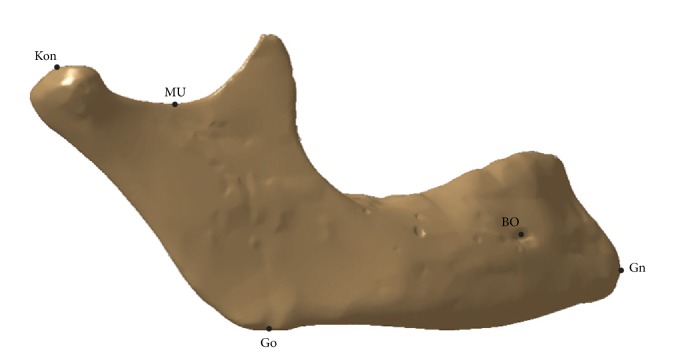
Anatomical landmarks (points).

The characteristic anatomical landmarks (points in this case) defined on the mandible are shown in Table 1 and described in [22] by Arsic et al. The mandibular cut (MU) was added by the authors of this research, because it was necessary to add this point as additional support point for the proper definition of coronoid process geometry. The points shown in Figure 3 and described in Table 1 are defined as RGEs used for the creation of the parametric model of the Human Mandible Coronoid Process.

### 2.3. Morphometric Parameters of the Human Mandible

**Figure 4:**
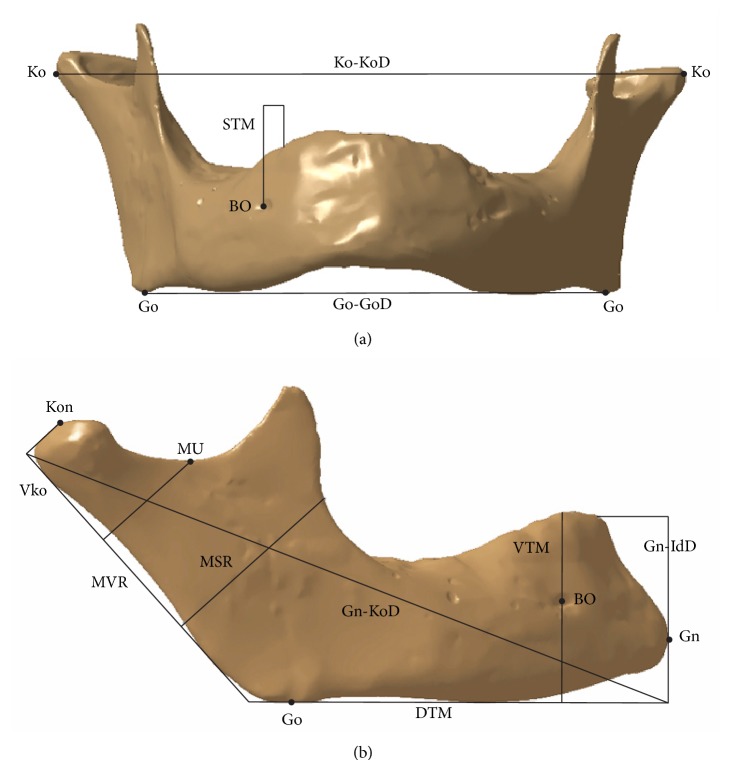
Morphometric parameters and anatomical points presented on polygonal model of mandible.

There are two groups of morphometric parameters: linear (lines, planes, and points) and angular (defining relative position of mandible parts). The values of mandible morphometric parameters can define sex, some irregularities in the skeletal system, parametric models, and so forth. The morphometric parameters were defined as geometrical elements on the polygonal model of the human mandible. As it is stated in [22], ten morphometric parameters are enough for the complete definition of mandible geometry, and they are defined in Table 2 and presented in Figure 4.

### 2.4. The Parametric Model of Human Mandible Coronoid Process

The first step in definition of the parametric model is the creation of the model coordinate system which is used for the measurement of the coordinates of the anatomical points. The same coordinate system is created on each polygonal model of individual mandible.

**Figure 5:**
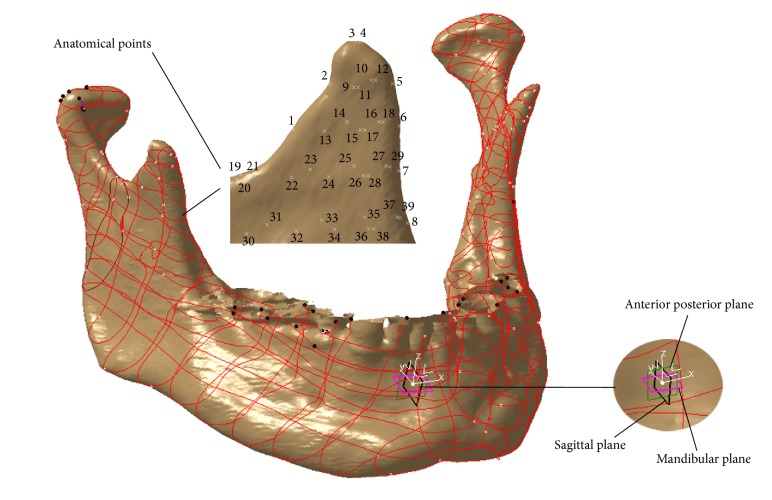
Coordinate system, spline curves, and anatomical points defined on human mandible polygonal model.

Origin of the coordinate system is defined as the middle of the distance between mental foreman middle points. The constructed planes of the Object Coordinate System (OBC) of mandible are presented in Figure 5 and they are Mediosagittal, mandibular, and coronal. The Mediosagittal (MS) plane was constructed as the plane which contains the origin of the OBC, and it is normal to the line which connects mental foreman middle points. MS is a plane which divides human mandible into two halves, left and right. Horizontal or mandibular plane is the lowest plane normal to the MS plane and it contains the gonion anatomical point (the most inferior point of the symphysis of the mandible, as seen in the lateral jaw projection). To be used as a plane of OBC this plane was translated to the origin of OBC. Coronal or anterior posterior (AP) plane is a plane which is normal to the mandibular plane and divides mandible into two anatomical sections, anterior (front) and posterior (back). It is placed at the origin of OBC. *x*-axis of the OBC is defined as normal to MS plane. *y*-axis is defined as normal to AP plane. *z*-axis is normal to mandibular plane. Axes of the OBC are presented in Figure 5.

Coordinate system together with spline curves created on the polygonal model of mandible is shown in Figure 5. Spline curves are created by the intersection of defined planes (mandibular, sagittal, and coronal) and polygonal model of mandible. These curves are used as the basis for the creation of anatomical points, which were created on them. Some anatomical points were not created on spline curves, yet they were created directly on the polygonal model of the human mandible as additional support points.

The thirty-nine points were created in the area of the HMCP. The anatomical points are labeled so they represent some topological (e.g., curvature) and anatomical landmarks (e.g., point distinct from gonion) on the model. Their position was proposed by the orthodontist and anatomist involved in this research. Created anatomical points on the HMCP are presented in Figure 5.

On each individual model of mandible the values of coordinates of these points were measured (distance from origin of coordinate system in all three directions *X*, *Y*, and *Z*). These values were used as the input vectors for the multiple linear regression analysis, which is well known and documented statistical function. The morphometric parameters were also measured on each mandible sample and incorporated into regression functions. The multiple linear regression algorithm which is applied in this research is described in detail in [23] by Brown. The basic idea is to predict dependent variable *Y* which is based on the independent variable *X*. Using the least squares method, the best fitting line can be found by minimizing the sum of the squares of the distance from each data point on the line [23]. The basic model is presented in (1) and defined in [23]:

(1) $$\begin{array}{c} Y=XB+E, \end{array}$$

where  *Y* is dependent variable, *X* is independent variable, *B* is coefficients, and *E* is error variable.

The example of matrix equation for the coordinate of one point defined in Matlab is presented in

(2) Xcoord=−51.874−47.712−46.269−47.164−50.78−49.684−43.613′;d1=28.5531.90229.34229.22532.69731.72730.733′;d9=17.522.37524.28621.53322.68419.45416.193′;⋮d10=122.384136.216131.729124.194130.113116.171115.732′;X=onessized1d1d2d3d4d5d6d7d8d9d10;A=X′∗X;K=invA;B=A∖X′∗Xcoord;M=X∗B;E=Xcoord−M,

where  *X* coord is values of *X* coordinates for defined point, *d*1,…, *d*10 are measured values of defined parameters (arranged according to the order in Table 2), *B* is vector of the coefficients, *M* is values of the calculated coordinate *X*, *E* is the error vector, (measured values minus calculated values), and *A*, *K* are helper matrixes.

## 3. Results and Discussion

The calculation was performed for all thirty-nine points on the Human Mandible Coronoid Process. In Table 3 coefficients of the multiple linear regression functions are presented for four chosen anatomical points.

For example, statistical function for *X* coordinate of Point 1 is presented in

(3) Xb0+b1∗d1+b2∗d2+⋯+b10∗d10=>X=0.068+1.167∗d1−0.001∗d2+⋯−0.549∗d10.

By using these functions coordinates values were calculated and compared with measured values for all thirty-nine points. The maximal error for all coordinates for each individual patient is presented in Table 4. The maximum deviation is for *Y* coordinate of Point 8 shown in Figure 5. The orthodontists and anatomist suggested that maximal error in this area should not exceed two (2) mm in defined *X*, *Y*, and *Z* directions. Taking into account the orthodontists' recommendations it can be concluded that maximum deviations of the values of the coordinates are quite satisfactory and below recommended limit.

Maximum surface deviations of the surface model of HMCP created by the use of parametric functions (calculated model) from the input surface models of original mandible specimens are presented in Table 5. The surface models were created by the use of spline curves through the input and calculated points and with the application of technical features in CATIA software package (multisection surface, fill, etc.). It can be noticed that these values are also below the recommended limit. Maximal deviation is 1.36 mm.

The preliminary claim about parametric model geometrical accuracy and anatomical correctness can be stated as quite satisfactory for the prototype model. It is important to mention that designers can choose more points in the area of maximal deviation(s) or choose different points, which will enable better geometrical definition of the domain included and thus improve the accuracy.

In order to obtain reliable response of the parametric model, more detailed analysis must be performed. The number of samples should be increased, parameters influence on the individual points should be examined, and the parametric model for the whole human mandible must be created. These tasks will be conducted in the future research.

## 4. Conclusion

The presented Method of Anatomical Features (MAF) enables creation of the geometrically accurate and anatomically correct parametric model of the Human Mandible Coronoid Process (HMCP). The presented parametric model of the HMCP can be considered as a prototype (test) model for the parameterization of the whole human mandible.

It should be emphasized that parametric model enables creation of the adequate geometric model of the HMCP customized to the specific patient. The customization is performed by the application of the values of the parameters in the parametric functions. Values of the parameters can be acquired from medical images (CT, MRI, or X-ray). The resulting model(s) can be applied in training of the medical staff, implant and fixator manufacturing, CAD/CAM application, FEA (Finite Element Analysis), and so forth.

The current research results are based on a relatively small number of human mandible samples. It is crucial to increase that number as much as possible. Besides the number of samples, the influence of the involved parameters on the position of the individual points must be investigated. All of these tasks are the part of future research and they will be performed in order to improve the geometric precision and anatomical correctness of the presented parametric model of the HMCP and future parametric model of the whole human mandible. One possible application of the parametric model of the whole human mandible is for the prediction of the dental implants position and orientation. For example, it can be used for the proper implantation of the osseointegrated dental implants which are presented in [24] by Vairo and Sannino. Considering that and all other facts presented in this paper, it can be concluded that further research is advisable because it can provide a lot of benefits to the medical practitioners.

## Figures and Tables

**Table 1:** Anatomical landmarks (points) defined on the human mandible as RGEs.

Anatomical landmark	Definition
Mental foramen (BO)	Is one of two holes (“foramina”) located on the anterior surface of the mandible?
Gnathion (Gn)	It is the most inferior midline point on the mandible.
Gonion (Go)	It is a point along the rounded posteroinferior corner of the mandible between the ramus and the body.
Condylion (Kon)	It is the most prominent point on the mandibular condyle.
Mandibular cut (MU)	It is the central point on the mandibular notch.

**Table 2:** Morphometric parameters of the human mandible.

Morphometric parameters	Definition
Gnathion-interdental distance (Gn-IdD)	Direct distance from infradentale (idD) to gnathion (Gn).
Bigonial width (Go-GoD)	Direct distance between right and left gonion (Go).
Bicondylar breadth (Ko-KoD)	Direct distance between the most lateral points on the two condyles.
Height of the mandibular body (VTM)	Direct distance from the alveolar process to the inferior border of the mandible perpendicular to the base at the level of the mental foramen.
Breadth of the mandibular body (STM)	Maximum breadth measured in the region of the mental foramen perpendicular to the long axis of the mandibular body.
Mandibular length (DTM)	Distance of the anterior margin of the chin from a center point on the protected straight line placed along the posterior border of the two mandibular angles.
Minimum ramus breadth (MSR)	Least breadth of the mandibular ramus measured perpendicular to the height of the ramus.
Maximum ramus height (MVR)	Direct distance from the highest point on the mandibular condyle to gonion (Go).
Height of the condyles (VKo)	Distance between Kon and axis of the lowest point of mandibular cut perpendicular to MVR.
Gnathion-condylar distance (Gn-KoD)	Distance between Gn and Kon.

**Table 3:** Coefficients of the multiple linear regression functions for four anatomical points.

Point		*b*0	*b*1	*b*2	*b*3	*b*4	*b*5	*b*6	*b*7	*b*8	*b*9	*b*10
P1	*x*	0.068	1.167	−0.001	−0.685	2.443	−0.648	−0.096	0.362	−0.102	0.317	−0.549
	*y*	24.840	0.738	−0.002	−0.650	1.732	−0.104	0.235	0.784	0.952	−0.471	−0.402
	*z*	18.606	0.161	0.000	−0.325	1.247	−0.113	0.080	−0.330	0.753	−0.582	−0.140

P2	*x*	12.708	1.115	−0.001	−0.790	3.060	−0.891	−0.012	0.181	−0.162	0.192	−0.621
	*y*	13.340	1.226	−0.002	−0.773	1.644	0.044	0.211	0.969	1.170	−0.565	−0.449
	*z*	18.989	0.095	0.000	−0.318	1.393	−0.221	0.004	−0.140	0.819	−0.387	−0.173

$\begin{array}{c} \vdots \end{array}$	$\begin{array}{c} \vdots \end{array}$	$\begin{array}{c} \vdots \end{array}$	$\begin{array}{c} \vdots \end{array}$	$\begin{array}{c} \vdots \end{array}$	$\begin{array}{c} \vdots \end{array}$	$\begin{array}{c} \vdots \end{array}$	$\begin{array}{c} \vdots \end{array}$	$\begin{array}{c} \vdots \end{array}$	$\begin{array}{c} \vdots \end{array}$	$\begin{array}{c} \vdots \end{array}$	$\begin{array}{c} \vdots \end{array}$	$\begin{array}{c} \vdots \end{array}$

P38	*x*	−6.762	1.098	−0.001	−0.519	1.454	−0.591	−0.044	0.335	−0.107	0.087	−0.378
	*y*	18.717	1.684	−0.002	−1.030	2.287	0.181	0.386	0.764	1.158	−0.870	−0.576
	*z*	−0.679	0.276	0.000	−0.105	0.121	0.008	0.021	−0.405	0.657	−0.554	0.046

P39	*x*	−3.095	1.215	−0.001	−0.605	1.388	−0.470	0.077	0.536	−0.062	−0.107	−0.409
	*y*	17.478	1.715	−0.002	−1.018	2.109	0.210	0.413	0.773	1.160	−0.943	−0.551
	*z*	−0.669	0.276	0.000	−0.105	0.122	0.008	0.021	−0.405	0.657	−0.553	0.046

**Table 4:** The maximal error for all coordinates of anatomical points for each individual patient.

Coord. [mm]	Pat. 1	Pat. 2	Pat. 3	Pat. 4	Pat. 5	Pat. 6	Pat. 7	Pat. 8	Pat. 9	Pat. 10
*X*	0.980	0.732	0.448	0.227	0.227	0.775	0.995	0.92	0.216	0.897
*Y*	**1.720**	0.668	0.597	0.453	0.907	0.469	0.952	0.765	0.301	0.842
*Z*	0.554	0.256	0.567	0.991	0.178	0.928	0.370	0.721	0.650	0.461

**Table 5:** Maximum deviations of the calculated surface model of the HMCP from the input HMCP models.

Model	1	2	3	4	5	6	7	8	9	10
Max. deviation [mm]	**1.36**	0.526	0.661	0.654	0.97	0.912	1.3	0.97	0.34	0.87
